# Phytochemicals from *Kaempferia angustifolia* Rosc. and Their Cytotoxic and Antimicrobial Activities

**DOI:** 10.1155/2014/417674

**Published:** 2014-06-25

**Authors:** Sook Wah Tang, Mohd Aspollah Sukari, Bee Keat Neoh, Yunie Soon Yu Yeap, Ahmad Bustamam Abdul, Nurolaini Kifli, Gwendoline Cheng Lian Ee

**Affiliations:** ^1^Department of Chemistry, Faculty of Science, Universiti Putra Malaysia, 43400 Serdang, Selangor, Malaysia; ^2^Sime Darby Technology Center, 2 Jalan Tandang, 46050 Petaling Jaya, Selangor, Malaysia; ^3^UPM-MAKNA Cancer Research Laboratory, Institute of Bioscience, Universiti Putra Malaysia, 43400 Serdang, Selangor, Malaysia; ^4^Pangiran Anak Puteri Rashidah Sa'adatul Bolkiah Institute of Health Science, Universiti Brunei Darussalam, Jalan Tungku Link, Gadong BE1410, Brunei Darussalam

## Abstract

Phytochemical investigation on rhizomes of *Kaempferia angustifolia* has afforded a new abietene diterpene, kaempfolienol (**1**) along with crotepoxide (**2**), boesenboxide (**3**), 2′-hydroxy-4,4′,6′-trimethoxychalcone (**4**), zeylenol (**5**), 6-methylzeylenol (**6**), (24*S*)-24-methyl-5*α*-lanosta-9(11), 25-dien-3*β*-ol (**7**), sucrose, *β*-sitosterol, and its glycoside (**8**). The structures of the compounds were elucidated on the basis of spectroscopic methods (IR, MS, and NMR). Isolation of 6-methylzeylenol (**6**), (24*S*)-24-methyl-5*α*-lanosta-9(11), 25-dien-3*β*-ol (**7**), and *β*-sitosterol-3-*O*-*β*-D-glucopyranoside (**8**) from this plant species has never been reported previously. The spectroscopic data of (**7**) is firstly described in this paper. Cytotoxic screening indicated that most of the pure compounds tested showed significant activity with (**4**) showing the most potent activity against HL-60 (human promyelocytic leukemia) and MCF-7 (human breast cancer) cell lines. However, all extracts and most of the pure compounds tested were found to be inactive against HT-29 (human colon cancer) and HeLa (human cervical cancer) cell lines. Similarly, none of the extracts or compounds showed activity in the antimicrobial testing.

## 1. Introduction

*Kaempferia *is a genus of herbs of over 50 species natives of east tropical Asia belonging to the Zingiberaceae family. It is small rhizomatous herb with usually thick aromatic tuberous roots and short rhizomes.* Kaempferia angustifolia* is one of the* Kaempferia *species which is less well known in Malaysia, if compared with* K. galanga *and* K. rotunda*. Itis locally known as* Kunci pepet, Kunci menir, *or* Kunci kunot*. This plant is tuberous and stemless, has small leaves, and could be found growing wildly in the forests of west and center of Java. It has nice smell and is usually used as medicine to treat cold, stomach-ache, and dysentery, while its rhizome is used for coughs and as a masticatory [1].

In recent years, many researchers have indicated the high potential of edible Southeast Asia plants for cancer chemoprevention and as antimicrobial agents. Zingiberaceae have been found to be one of the desirable sources of effective cancer-preventive agents since Zingiberaceous plants demonstrated promising inhibitory effect on the growth of human breast cancer (MCF-7), colon cancer (HT-29 and Col2), lung cancer (A549), stomach cancer (SNU-638), and cervical cancer (CaSki) cell lines [2–4]. Besides, antimicrobial activities of ginger were also a topic of interest. Reports on the screening of extracts or essential oils of members from Zingiberaceae family against bacterial strains, fungi, and yeast have been published [5–7].* Alpinia*,* Curcuma,* and* Zingiber *species were frequently studied but literature on antimicrobial properties of* Kaempferia* species was not studied as much. Essential oil of* Kaempferia angustifolia* has been reported [8] to have moderate antimicrobial activity towards inhibition of* Staphylococcus aureus* and* Pseudomonas aeruginosa* (10.63 ± 0.23, 10.03 ± 0.22 mm, resp.) but not against* Escherichia coli*,* Bacillus subtilis*,* Streptococcus faecalis*,* Candida albicans,* and* Microsporum gypseum*.

Phytochemical investigations of* Kaempferia* species have yielded several compounds including cyclohexane diepoxide derivatives, flavonoids, and diterpenes [9–11]. Most of the consumable ginger rhizomes have long been used as folk medicine for centuries but have not been thoroughly investigated for their bioactive properties yet. Thus, here we wish to report the isolation and cytotoxic and antimicrobial properties of chemical constituents from rhizomes of* Kaempferia angustifolia*.

## 2. Materials and Methods

### 2.1. General Experimental Procedures

Melting points (uncorrected) were determined using Barnstead Electrothermal IA 9100 Series melting points equipment. Optical rotations were measured on a Jasco P-2000 polarimeter equipped with a sodium lamp (589 nm). The IR spectra were recorded using KBr discs on Perkin Elmer FTIR spectrophotometer 1650. ^1^H and ^13^C NMR spectra were obtained on JEOL spectrometer at 500 and 125 MHz and/or 400 and 100 MHz, respectively, with tetramethylsilane (TMS) as internal standard. Mass spectra were recorded on Shimadzu model QP 5050 A spectrometer. Ultraviolet spectra were recorded on a Shimadzu UV-1601 spectrophotometer. Separation by column chromatography was carried out by using silica gel (Merck 7749, 7734, and 9385), while silica gel 60 PF_254_ aluminium sheets were used for TLC analysis.

### 2.2. Plant Material and Cytotoxic Cell Lines

*Kaempferia angustifolia *was collected from Java, Indonesia, in 2001. The plant was identified by Dr. Sugeng Riyanto, Faculty of Pharmacy, Gadjah Mada University, and the voucher specimen (number 16/SR/0601-01) was deposited at the herbarium of the institution. The HL-60 (human promyelocytic leukemia), MCF-7 (human breast cancer), HT-29 (human colon cancer), and HeLa (human cervical cancer) cell lines were obtained from the National Cancer Institute, Maryland, USA. Bacterial strains were originally obtained from American Type Cell Culture Collection (ATCC), USA.

### 2.3. Extraction and Isolation

The finely ground air-dried rhizomes (1.0 kg) were extracted repeatedly with petroleum ether, chloroform, and methanol thrice each for 72 hours. The extracts were filtered and concentrated under reduced pressure to obtain petroleum ether (8.40 g), chloroform (7.50 g), and methanol (6.70 g) extracts. The petroleum ether extract was triturated with methanol at room temperature to give a solid product which was then filtered and recrystallized in methanol to yield crotepoxide (**2**) (16 mg). Brown oil extract (5.80 g) was obtained upon evaporation of the filtrate. The extract was subjected to vacuum column chromatography and eluted stepwise with 100% hexane, hexane-ethyl acetate, ethyl acetate-methanol, and 100% methanol. This afforded boesenboxide (**3**) (40 mg), 2′-hydroxy-4, 4′, 6′-trimethoxychalcone (**4**) (26 mg), and *β*-sitosterol (18 mg). Spectroscopic data of compounds** 2**–**4** have been reported previously [12]. Chloroform extract was subjected to column chromatography separation and eluted with mixture of hexane and ethyl acetate to give 23 fractions (200 mL each). Fraction three which was eluted from hexane-ethyl acetate (5 : 5) was purified to afford zeylenol (**5**) (0.238 g), while fraction four obtained from elution with mixture of hexane-ethyl acetate (3 : 7) was rechromatographed over silica gel to give subfractions 17–19 which were then recrystallized with methanol to afford 6-methylzeylenol (**6**) (42 mg). Methanol extract was fractionated using column chromatography in a similar manner. Eighteen fractions of 200 mL each were collected. Combination and purification of fractions 14 and 15 from elution with ethyl acetate-methanol (7 : 3) gave sucrose (11 mg). A reinvestigation of the sample (765.0 g) from the same source was conducted by extraction with methanol (3 × 72 hours) to obtain a dark brown viscous liquid extract which was then partitioned with hexane and water with 10% methanol (1 : 1) to give hexane-soluble fraction (10.60 g) as brown yellowish viscous liquid. The hexane fraction (8.00 g) was subjected to column chromatography eluted with mixtures of hexane, ethyl acetate, and methanol of increasing polarity to give 86 fractions (200 mL each). Upon washing and recrystallizing with methanol, fractions 13-4 which were eluted from hexane : ethyl acetate (97 : 3) afforded (24*S*)-24-methyl-5*α*-lanosta-9(11) and 25-dien-3*β*-ol (**7**) (40 mg). Fraction 84 which was eluted from ethyl acetate : methanol (50 : 50) was further washed with methanol to give *β*-sitosterol-3-*O*-*β*-D-glucopyranoside (**8**) (8 mg). Similarly, compound** 2** (10 mg), compound** 4** (7 mg), and *β*-sitosterol (35 mg) were also obtained from this extract. Chloroform-soluble fraction (40.000 g) was fractionated in a similar manner to obtain 100 fractions (200 mL each) which were combined into major fractions based on their TLC profiles. Compounds** 1** (20 mg),** 2 **(0.79 g),** 3 **(16 mg),** 4 **(10 mg), and** 5** (0.97 g) were subsequently obtained. Isolation procedures and spectroscopic data of compounds** 1** and** 5 **have been reported previously [13].

### 2.4. Spectral Data

(24*S*)-Methyl-5*α*-lanosta-9(11),25-dien-3*β*-ol (7); C_31_H_52_O, white needle-shaped crystal. Melting point: 180-181°C. [*α*]_*D*_: +67.5(*c* 0.04, CHCl_3_). *R*_*f*_: 0.60 (Hexane-EtOAc, 4 : 1). IR (KBr): 3684, 3402, 2940, 1644, 1098, 888 cm^−1^. UV/Vis *λ*_max⁡_ (CHCl_3_) nm (log⁡*ε*): 239.2 (3.837). ^1^H NMR (400 MHz, CDCl_3_) and ^13^C NMR (100 MHz, CDCl_3_): Table 1. EI-MS: *m*/*z*(%) = 440 (M^+^, 27), 425 (56), 407 (31), 313 (100), 69 (72), 55(81).

### 2.5. Cytotoxic Assay

The cytotoxic assay was carried out according to the methods described previously [14]. Cytotoxic and proliferation profiles were determined by MTT (3-[4,5-dimethylthioazol-2-yl]-2,5-diphenyltetrazolium bromide) assay whereby cell viability of treated cell population was compared to the control cell population. The cell viability was determined by the formation of blue formazan crystals of MTT in which the crystals were dissolved in DMSO and the absorbance was read with Elisa reader test wavelength of 570 nm and references wavelength of 630 nm. Four human cancer cell lines which included HL-60 (human promyelocytic leukemia), MCF-7 (human breast cancer), HT-29 (human colon cancer), and HeLa (human cervical cancer) cell lines were tested against crude extracts and pure compounds isolated. A graph of percentage of cell viability versus the concentration of crude extracts or pure compounds tested was plotted and the IC_50_ values were determined. The cytotoxic index used was IC_50_, which is the concentration that yields 50% inhibition of the treated cell compared with untreated control. Extracts that show IC_50_ < 30 *μ*g/mL are considered to have significant cytotoxic activity whilst pure compounds that show cytotoxicity IC_50_ < 20 *μ*g/mL are qualified for further cytotoxicity investigations [15].

### 2.6. Antimicrobial Assay

The extracts and isolated compounds were subjected to antimicrobial testing against several microbes including methicillin resistant* Staphylococcus aureus*,* Pseudomonas aeruginosa* (ATCC 60690),* Salmonella choleraesuis,* and* Bacillus subtilis *(wild) (B_29_) for antibacterial screening and* Candida albicans*,* Saccharomyces cerevisiae,* and* Aspergillus ochraceus* (ATCC 398) for antifungal screening using disc diffusion method as described in [16]. Ampicillin (Gram-negative bacteria) and streptomycin (Gram-positive bacteria) standards were used for each of the bacteria whilst nystatin was used for fungi and yeast as positive control. The antimicrobial activity was evaluated qualitatively by measuring the diameter of the clear inhibition zones around the impregnated discs. The activity index was determined by dividing its zone of inhibition by the standard antibacterial agent. The samples that show activity of >0.5 were considered to exhibit significant antibacterial activity [17].

## 3. Results and Discussion

**Figure 1:**
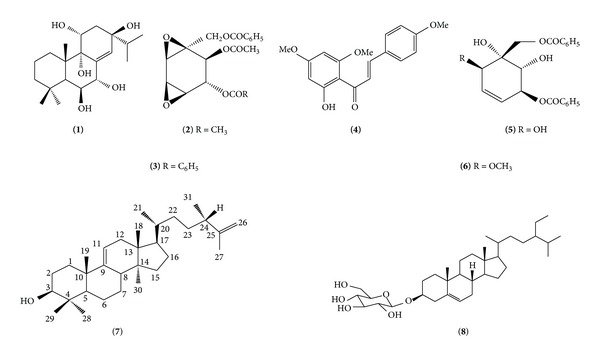
Structures of chemical constituents from* Kaempferia angustifolia*, kaempfolienol (**1**), crotepoxide (**2**), boesenboxide (**3**), 2′-hydroxy-4,4′,6′-trimethoxychalcone (**4**), zeylenol (**5**), 6-methylzeylenol (**6**), (24*S*)-24-methyl-lanosta-9(11), 25-dien-3*β*-ol (**7**), and *β*-sitosterol-3-*O*-*β*-D-glucopyranoside (**8**).

Extraction and separation on the isolates of various extracts of* K. angustifolia* led to the isolation and characterization of kaempfolienol (**1**), crotepoxide (**2**), boesenboxide (**3**), 2′-hydroxy-4,4′,6′-trimethoxychalcone (**4**), zeylenol (**5**), 6-methylzeylenol (**6**), (24*S*)-24-methyl-5*α*-lanosta-9(11), and 25-dien-3*β*-ol (**7**), together with sucrose, *β*-sitosterol, and its glycoside (**8**) (Figure 1). To the best of our knowledge, compounds** 6**,** 7, **and** 8** were isolated for the first time from this plant species. In this report, we describe the spectroscopic data of compound** 7** as well as cytotoxic and antimicrobial activities of the plant constituents. The details of the isolation and characterization of new compound, kaempfolienol (**1**), zeylenol (**5**), and its derivatives have been described in our previous report [13]. The compounds were characterized using spectroscopic methods and by comparison with the literature [12, 18–21].

Compound** 6** (0.042 g) with molecular formula of C_22_H_22_O_7_ was obtained as colourless needle-shaped crystals with melting point of 206-207°C. The FAB-MS spectrum showed the [M+H]^+^ peak at* m/z* 399 which corresponds to the molecular formula mentioned. All spectral data were in accordance with the previously reported data of compound** 6** which is isolated from* Uvaria purpurea* [14].

**Figure 2:**
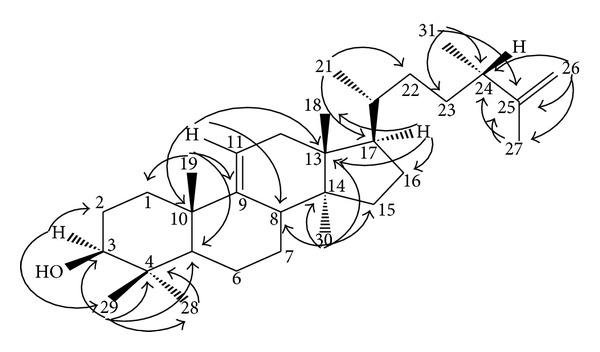
Selected HMBC correlations of (**7**).

Compound** 7** was isolated as white needle-shaped crystal with melting point of 180-181°C. The EI-MS spectrum showed a molecular ion at* m/z* 440 corresponding to molecular formula of C_31_H_52_O, indicative of six degrees of unsaturation. The IR spectrum of (**7**) indicated the presence of hydroxyl group at 3684 and 3402 cm^−1^ and olefinic functional group at 1644 cm^−1^. The ^1^H NMR spectrum demonstrated signals for six tertiary methyl groups and two secondary methyl groups ranging from *δ* 0.64 to 1.64, three olefinic protons at *δ* 4.66 (2H) and 5.22 (1H), and a deshielded methine proton at *δ* 3.22. These spectral data (Table 1) suggested that compound** 7** is an unsaturated triterpene alcohol. The ^13^C NMR experiments including DEPT sorted 31 signals into eight methyls, ten methylenes, six methines, and seven quaternary carbons. The carbon signals for (**7**) were almost identical to the corresponding carbon signals of reported data for its acetyl derivative [20] except for C-2 to C-4 and the signals for 4*β*-methyl (C-29). The chemical shifts values of C-3, C-2, and C-4 revealed an upfield shift of 2.0 ppm and a downfield shift of 3.6 and 1.1 ppm, respectively, compared to those of corresponding data of (24*S*)-24-methyl-5*α*-lanosta-9(11), 25-dien-3*β*-acetate [20]. It was thus clear that a hydroxyl group was located at C-3. The precise NMR signals connectivities of (**7**) were deduced by interpretation of the significant HMBC spectrum and are shown in Figure 2. The olefinic proton at *δ* 5.22 (*d*, 6.4 Hz) was correlated with C-10, C-13, and C-8 via ^3^J correlation and, hence, the double bond was placed at C-9/C-11. Two exomethylene protons which resonated as singlet at *δ* 4.66 exhibited a* w*-coupling to the deshielded methyl group at *δ* 1.64 (H-27) in COSY spectrum and were correlated to C-24, C-25, and C-27 in HMBC spectrum. On the basis of the above results, compound** 7** was characterized as (24*S*)-24-methyl-5*α*-lanosta-9(11), 25-dien-3*β*-ol. This is the first isolation of the compound as triterpene alcohol since previous literature reported it as its acetyl derivatives isolated from* Glycosmis arborea* and* Neolitsea aciculata *[20, 21].

Compound** 8** was obtained as white amorphous powder from the last fraction of hexane extract. The molecular ion peak for compound** 8** which is suggested to be at* m/z* 576 could not be traced in the EI-MS spectrum. However, the fragment ions at* m/z* 414 [M-C_6_H_10_O_5_]^+^, 396 [M-C_6_H_10_O_5_-H_2_O]^+^, 255 [M-C_6_H_10_O_5_-H_2_O-C_10_H_21_(side chain)]^+^, and* m/z* 127, 73, and 57 which were obtained from the sugar moiety suggested that compound** 8** may be monoglycoside with a C_29_-sterol (*m/z* 414) aglycone moiety. Further analysis of FAB-MS spectrum demonstrated the molecular ion peak at 599 [M+Na]^+^ which supported the molecular formula of C_35_H_60_O_6_. From the evidences in spectral data and comparison with reported data [19], compound** 8 **was, therefore, established as *β*-sitosterol-3-*O*-*β*-D-glucopyranoside which was isolated previously from* Prunella vulgaris*.

The cytotoxic screening results (Table 2) suggested the antagonist effect shown by the isolated compounds of* Kaempferia angustifolia* against HL-60 (human promyelocytic leukemia), MCF-7 (human breast cancer), and HeLa (human cervical cancer) cell lines. All crude extracts tested were inactive against all cell lines tested, with IC_50_ > 30 *μ*g/mL. Among the cell lines examined, HL-60 and MCF-7 cells exhibited high sensitivity for all tested compounds (**1**–**8**), except for the weak activity shown by compound** 2** against HL-60 cell and compound** 5** towards MCF-7. Most of the isolated compounds were inactive against HT-29 (human colon cancer) and HeLa cell lines, with the exception of compound** 7** which showed strong activity with IC_50_ of 1.4 *μ*g/mL, which is comparable to standard (5-fluorouracil). Compounds** 6** and** 8** also showed moderate inhibition against HeLa cell line. Extracts or isolated compounds, which give IC_50_ < 10 *μ*g/mL, were considered to have significant cytotoxic activity [19]. Cytotoxic agents that have been used as standard (positive control) in the screening test were tamoxifen, goniothalamin, and 5-fluorouracil.

Interestingly, 2′-hydroxy-4,4′,6′-trimethoxychalcone (also known as flavokawain A) (**4**) appeared to be the most potent compound in cytotoxic screening against both HL-60 and MCF-7 cell lines. A recent publication [22] investigated the antiproliferative effects of this compound against a panel of cancerous cell lines. The results revealed that flavokawain A (**4**) exhibited similar cytotoxic property against MCF-7 cell line but demonstrated better anticancer effect against HT-29 cell line as compared with our findings, with GI_50_ values of 17.5 *μ*M (5.5 *μ*g/mL) and 45.3 *μ*M (14.2 *μ*g/mL), respectively. The presence of methoxy substituents and *α*-methylation of the enone moiety and the presence of 2′ oxygenated substituents are favourable structural requirements for cytotoxic activity due to antimitotic activity while several ring-A methoxylated chalcones exhibited cytotoxic activity by inhibiting tubulin polymerization against a variety of tumor cell lines in the low micromolar range (IC_50_ < 50 *μ*M) [23]. Kaempfolienol (**1**) showed moderate inhibition against HL-60 and MCF-7 cell lines with IC_50_ < 30 *μ*g/mL while zeylenol (**2**) demonstrated moderate inhibition towards HL-60 only with IC_50_ of 11.6 *μ*g/mL as reported previously [13].

As for antimicrobial testing, crude extracts and constituents of* Kaempferia angustifolia* were assayed against Gram-positive* Bacillus subtilis*, MRSA, Gram-negative* Pseudomonas aeruginosa*,* Salmonella choleraesuis*,* Candida albicans* (yeast), and fungi* Aspergillus ochraceus* and* Saccharomyces cerevisiae*. However, no activity was detected for all the samples against all microbes tested. Compounds (**4**) and (**6**) were not tested towards* C. albicans* and fungi due to insufficient amount of samples. The antifungal properties of flavokawain A (**4**) against* Saccharomyces cerevisiae*,* Candida albicans, *and some other fungi were reported to be inactive [24], and these results complement our findings. Compound** 4** did not show any inhibition towards Gram-positive and Gram-negative bacteria tested in this study.

The isolation of triterpene from* Kaempferia* species is unprecedented besides reports on common plant sterols such as *β*-sitosterol and stigmasterol. The physical properties and spectral data of (24*S*)-24-methyl-5*α*-lanosta-9(11), 25-dien-3*β*-ol (**7**) are firstly described in this paper. The biotaxonomic distribution of lanostanes is quite restricted [25]. They are abundant in Polyporaceae and other fungi but also appear in some Spermatophyta like Orchidaceae, Pinaceae, and Anacardiaceae. The isolation of lanostane-type triterpene (compound** 7**) from Zingiberaceae has never been reported before and, hence, this finding has contributed to the chemotaxonomic significance.

## 4. Conclusion

The rhizome extracts of* Kaempferia angustifolia *Rosc. afforded eight compounds (terpene, triterpene, cyclohexane derivatives, chalcone, and glycoside) which showed cytotoxicity against some human cancer cell lines. The structures of the isolates were identified and elucidated based on spectroscopic method and comparison of literature reviews.

## Figures and Tables

**Table 1:** ^
1^H (400 MHz) and ^13^C-NMR (100 MHz) spectral data of compound **7** (CDCl_3_).

Position	*δ* ^ 1^H (ppm) (multiplicity, *J* in Hz)	*δ* ^ 13^C (ppm)
1	1.35 (m), 1.45 (m)	36.1
2	1.67 (m)^a^, 1.72 (m)	27.8
3	3.22 (dd, 11.9, 4.6)	78.9
4	—	39.1
5	0.87 (d, 6.4)^b^	52.5
6	1.47 (dd, 12.9, 2.7), 1.68 (m)^a^	21.4
7	1.30 (m)	28.1
8	2.16 (m)	41.8
9	—	148.5
10	—	39.4
11	5.22 (d, 6.4)	115.0
12	1.91 (br d, 6.4),	37.1
	2.08 (br d, 6.4)	
13	—	44.3
14	—	47.0
15	1.33 (m)^c^	33.9
16	1.87 (br d, 4.6)	27.9
17	1.57 (m)	50.9
18	0.64 (s)	14.4
19	1.04 (s)	22.3
20	1.80 (m)	36.0
21	0.87 (d, 6.4)^b^	18.4
22	1.33 (m)^c^	34.0
23	1.15 (m), 1.42 (m)	31.4
24	2.10 (m)	41.6
25	—	150.2
26	4.66 (s)	109.4
27	1.64 (s)	18.6
28	0.99 (s)	28.2
29	0.82 (s)	15.7
30	0.73 (s)	18.5
31	1.00 (d, 7.4)	20.2

^a,b,c^Overlapped signals.

**Table 2:** IC_50 _values for pure compounds of *K. angustifolia* against HL-60, MCF-7, HT-29, and HeLa cell lines.

Samples	∗IC_50_ (*μ*g/mL)			
	HL-60	MCF-7	HT-29	HeLa
**1**	24.22 ± 0.30	23.50 ± 1.70	>30	>30
**2**	>30	18.09 ± 0.20	>30	>30
**3**	7.77 ± 0.48	19.23 ± 0.69	>30	>30
**4**	7.49 ± 1.24	6.24 ± 0.57	>30	>30
**5**	11.65 ± 0.52	>30	>30	>30
**6**	19.63 ± 0.51	17.63 ± 0.84	—	19.45 ± 0.50
**7**	6.10 ± 0.22	5.75 ± 1.54	>30	1.42 ± 0.16
**8**	23.09 ± 3.39	19.83 ± 1.15	>30	23.95 ± 0.36
Standard goniothalamin tamoxifen 5-fluorouracil	1.4	2.8	4.2	1.2

*Values were means ± standard deviation of triplicate analyses.
